# Editorial: Neurophysiology in Alzheimer's Disease and Dementia

**DOI:** 10.3389/fnagi.2016.00153

**Published:** 2016-06-27

**Authors:** Davide V. Moretti

**Affiliations:** IRCCS, FBF, Rehabilitation in Alzheimer's Disease Operative Unit, S. John of God HospitalBrescia, Italy

**Keywords:** neurophysiology, EEG, alpha rhythm, magntoencefalography, Mismatch Negativity, mild cognitive impairment, Alzheimer's disease, genetics

The aging of the nervous system is often associated with chronic diseases typical of old age, which can offer in their pathogenesis, and in their susceptibility to particular therapies, the key to understanding the determinants of senescence. In recent years, the need to distinguish normal from pathological aging and the obligation to execute the diagnosis as early as possible, addressed dementia research to the field of biomarkers. The biomarkers are easily recognizable, quantifiable, and reproducible, biological entities, that can identify in a timely manner different profiles of disease. It is widely believed in the scientific community that the diagnosis of Alzheimer's disease (AD) can be made early in the integrated analysis of structural, biological, clinical, and functional biomarkers.

Neurophysiology, and in particular electroencephalography (EEG), has proved a reliable tool in the biomarkers research of dementias. The ability to highlight the state of the underlying brain network, even with very advance, the ease of application, the widespread reproducibility and, not least, the low cost of operation, makes this method very suitable for studies of with a large number of subjects.

In this special issue dedicated to the neurophysiology of dementia, contributions of different cultural origins are presented, contributing to the general richness, and the scientific significance of the issue.

Raymundo Cassani addressed the methodological aspect of removal of artifact from EEG, demonstrating that a wavelet enhanced independent component analysis (wICA) algorithm alone outperforms other methodics, thus opening the doors for fully-automated systems that can assist clinicians with early detection of AD, as well as disease severity progression assessment.

Francesco Di Lorenzo, addressed the basic neurophysiological aspect of the cholinergic system in AD through the theta burst stimulation (TBS) that modulate central cholinergic function using the neurophysiological determination of Short-Latency Afferent Inhibition (SLAI). The SLAI was decreased in AD patients compared to healthy controls (HS). Cerebellar TBS partially restored SLAI in AD patients but did not modify SLAI in healthy subjects. These results demonstrate that cerebellar magnetic stimulation affects cortical cholinergic activity and suggests that the cerebellum could have a direct influence on the cholinergic damage in AD.

A lot of studies addressed the clinical aspect of the early diagnosis in AD.

Christos A. Frantzidis demonstrated an impaired organization of brain networks even in the prodromal phase of Alzheimer's disease (AD) due, in hypothesis, to compensation mechanisms.

Zhijie Bian proposed that the theta/alpha EEG power band ratio recorded in the frontal and temporal region of the left hemisphere could be a diagnostic tool in patients with amnesic mild cognitive impairment (aMCI) affected by diabetes.

Simon-Shlomo Poil showed that EEG activity in the beta frequency range (13–30Hz) could be a helpful prognostic marker to predict the conversion from the MCI state to the overt AD condition. Of note, the authors found that the prediction power is higher (sensitivity of 88% and specificity of 82%) when EEG biomarkers are considered in an integrated way.

Davide V. Moretti demonstrated that the increase of EEG alpha3/alpha2 power ratio is related with prodromal phase of AD. The association with hippocampal atrophy, cortical thickness and reduction of brain regional cerebral perfusion suggests that alpha3/alpha2 power ratio could detect the MCI subjects who will convert to AD.

Very interestingly, Natalya Ponomareva confirmed the reliability of the increase of the EEG alpha3 spectral power investigating the age-related influence of the polymorphism of clusterin (CLU) genotype, associated with AD. In particular, the homozygous variant of CLU C allele is related with the increase of EEG alpha3 power, suggesting a possible susceptibility of this genetic variant carriers to hippocampal atrophy.

Monica Lindin explored the field of event-related potentials demonstrating that the Mismatch Negativity (MMN) amplitude was significantly smaller in aMCI subjects than in healthy controls.

Beyond EEG, also magnetoencephalography (MEG) could be a useful research tool. Pilar Garcés found a pathological alpha slowing in MCI patients when compared to healthy controls investigating the changes in the spatial distribution of the frequency and amplitude values of MEG alpha peak.

Finally, besides AD, Davide V. Moretti found that EEG frequency rhythms are sensible also to different stage of Fronto-Temporal-Dementia (FTD) and could detect changes in brain oscillatory activity affected by progranulin (GRN) mutations.

It is commonly thought that research in neuroscience is the researcher's effort to understand his own brain. If it is true, I want to thank all the authors for helping me to understand also something more of myself.

Somewhere, something incredible is waiting to be discovered (Cari Sagan)

## Author contributions

The author confirms being the sole contributor of this work and approved it for publication.

### Conflict of Interest Statement

The author declares that the research was conducted in the absence of any commercial or financial relationships that could be construed as a potential conflict of interest.

